# Dual-phase effects of emotional music on driving fatigue: delay and awakening revealed by pupillometry

**DOI:** 10.3389/fpsyg.2025.1647424

**Published:** 2025-09-17

**Authors:** Zhigang Hu, Kejin Li, Yi Cui, Xianru Shang

**Affiliations:** College of Design and Arts, Shaanxi University of Science & Technology, Xi'an, China

**Keywords:** fatigue driving, fatigue awakening, vocal stimulation, emotional music, eye movement behavior

## Abstract

This study investigated the effects of different emotional background music on driving fatigue through a 60-min simulated driving experiment. The research employed a dual-phase design (0–40 min fatigue-delaying phase and post-40 min fatigue-awakening phase) with three auditory conditions: positive music, negative music, and no background music (driving noise only). Pupil diameter variation coefficient and average blink rate were used as objective fatigue evaluation metrics. The results demonstrated that when different emotional background music was introduced before fatigue onset, positive music effectively delayed fatigue development during prolonged driving. Under monotonous driving noise conditions, positive music stimulation administered after fatigue onset significantly improved driver fatigue levels, with the most pronounced awakening effects observed when positive music followed negative music stimulation. These findings indicate that strategically selected positive background music can serve both preventive (delaying fatigue onset) and active (alleviating existing fatigue) functions, providing practical solutions for driver safety and fatigue management.

## 1 Introduction

Driving fatigue refers to the phenomenon that the driver's body dysfunctions after long-term continuous driving, thereby affecting the normal driving (Li et al., 2010). Research data show that fatigue driving is one of the important causes of traffic accidents (Tefft, 2012; Traffic Management Bureau of Ministry of Public Security, 2019). After the driver's brain is fatigued, drivers often fail to clearly recognize their brain fatigue and ignore it, which leads to accidents. Therefore, it is worth paying attention to the method of using reasonable methods to delay or awaken the driver's brain fatigue and restore the driver's good driving state.

Researches on driving fatigue is mainly divided into two aspects: prevention and countermeasures (Fletcher et al., 2005). The prevention refers to taking appropriate measures before the driver drives or develops fatigue to prevent or delay the occurrence of driving fatigue. The countermeasures are reflected in the driving process. When the driver is about to enter a state of fatigue or is already in driving fatigue, a certain awakening stimulus is given to prevent or relieve fatigue.

At present, the research on alleviating fatigue is mainly aimed at fatigue countermeasures, that is, taking fatigue awakening measures after the driver is fatigued. The awakening measures include setting visual fatigue wake-up belt (Li et al., 2021), intrusive wake-up, sound stimulation (Merat and Jamson, 2013), etc. Among them, sound stimulation is more usable due to its less restricted application. Many scholars' studies have shown that listening to music or radio after the driver is fatigued can relieve fatigue to a certain extent (Jin, 2021; Hirata, 2001; Gershon et al., 2011; Turner et al., 1996; Vogelpohl et al., 2019; Xiao and Zhu, 2021; Lu et al., 2016). Current research mainly focuses on the effects of music amplitude (Vogelpohl et al., 2019), pitch (Xiao and Zhu, 2021), rhythm (Lu et al., 2016), etc. on driving fatigue, and most of them only use one emotional type of music stimulation. This kind of musical stimulation is mostly introduced in the background of monotonous driving noise, and the long-term continuous stimulation will cause the driver to produce auditory adaptation, reduce the auditory sensibility, and the driver will still be fatigued after long-term driving.

To sum up, the current research has little discussion on whether music can effectively prevent or delay the occurrence of driving fatigue, whether different emotional types of music have different effects on fatigue, and whether the second introduction of music stimulation under different sound backgrounds can achieve fatigue awakening. Based on the above problems, this paper conducts a simulated driving test, introducing background music of different emotional types before fatigue occurs, and introducing sound stimuli again after fatigue occurs. To explore whether the introduction of back-ground music can delay the generation of driving fatigue, to clarify whether the second introduction of music stimulation has the effect of fatigue awakening, and then to obtain the awakening strategy to deal with driving fatigue.

As endogenous physiological signals, eye movement indicators are unaffected by external lighting interference. They can compensate for the environmental adaptability limitations of existing methods such as visual cameras and voice analysis, thereby enhancing the robustness of fatigue monitoring in complex scenarios. Eye movement metrics support localized personalized configuration, reducing reliance on cloud computing and mitigating attention risks caused by excessive entertainment features. This approach also meets high privacy requirements for driver fatigue monitoring. Therefore, research on driving fatigue using eye movement indicators holds significant importance in the current context.

## 2 Research purpose and content

**Figure 1:**
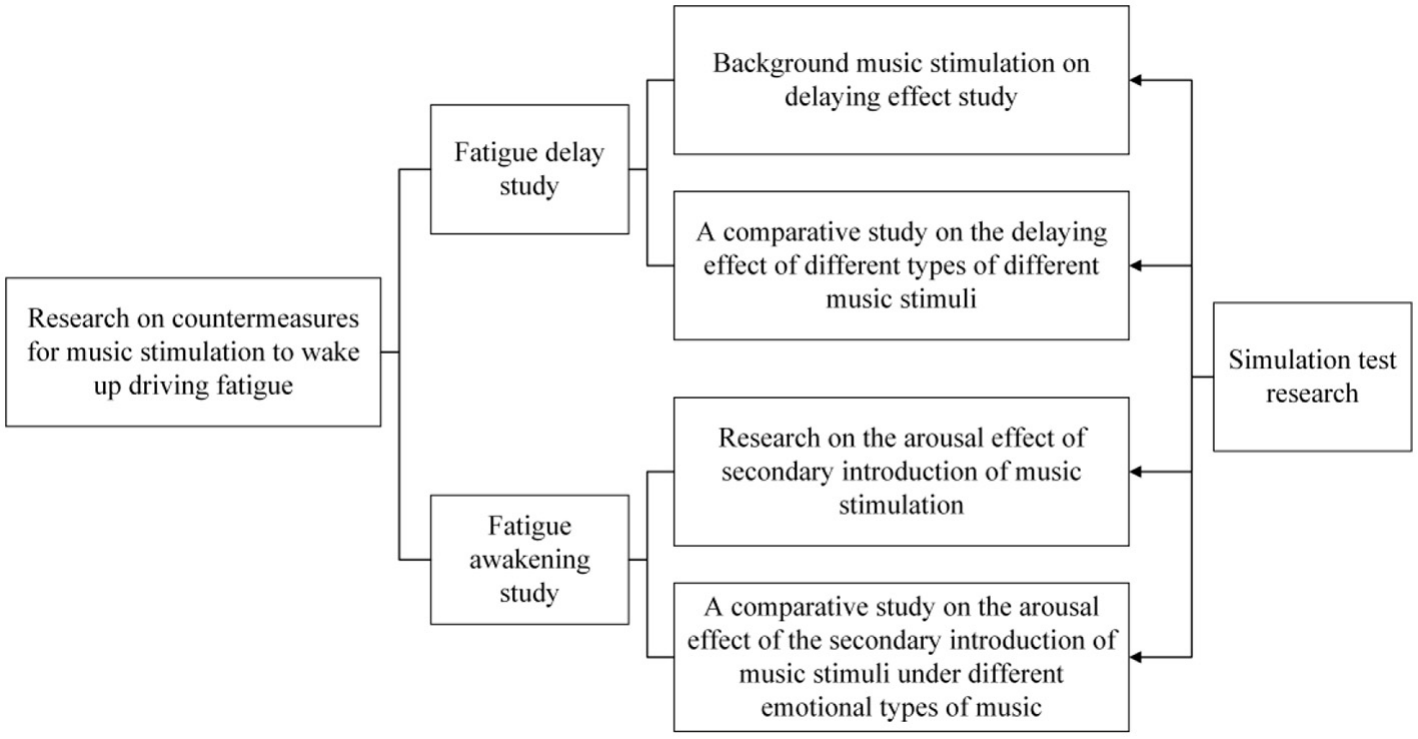
Research framework.

It is the main purpose to explore a music arousal strategy that can effectively relieve driving fatigue, mainly from two aspects of fatigue delay and fatigue awakening. In terms of fatigue delay, it is studied whether background music has a delaying effect on driving fatigue, and whether different emotional types of music have different de-laying effects on it. In terms of driving fatigue awakening, it mainly focuses on whether the secondary introduction of musical stimulation can produce fatigue awakening. In addition, to explore what kind of musical stimulation under what kind of musical background is better for fatigue awakening. The study framework is shown as Figure 1.

## 3 Experimental design and materials

### 3.1 Experimental design

**Figure 2:**
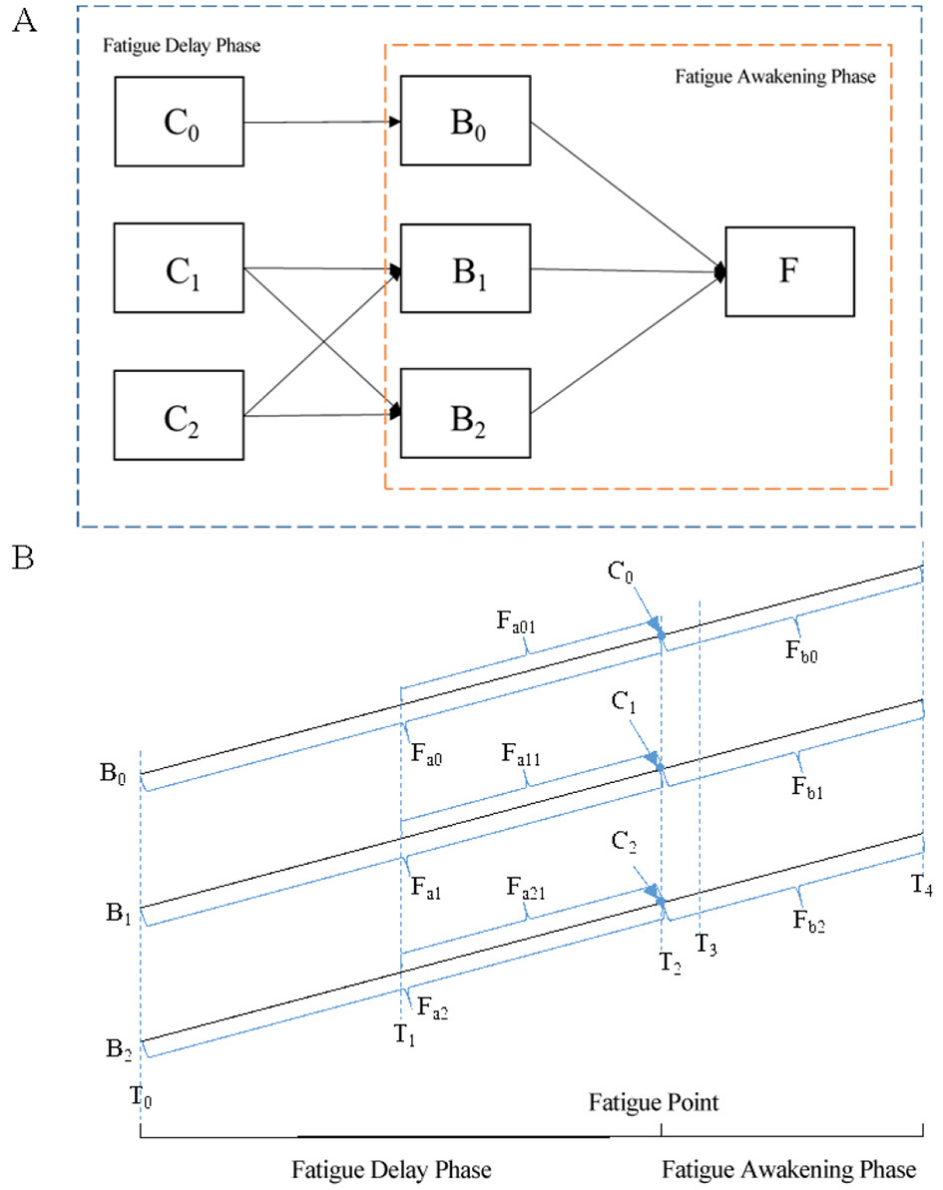
**(A)** Experiment design between factors model; **(B)** experiment design.

In order to explore the effects of different emotional types of background music and corresponding stimuli on driving fatigue, a 60-min simulated driving test was conducted using the controlled test method. The names and labels of the variables involved in the experiment are shown in Table 1, where the start of the experiment is recorded as T0, and the end of the experiment is recorded as T4. The between-factors model is shown in Figure 2A. The experiment is divided into fatigue delay phase and fatigue awakening phase. Referring to the other research (Gillberg et al., 1996; Mao et al., 2010), the object was judged to be in fatigue driving state after 40 min's driving. So, before 40 min, it was defined as the fatigue delay phase, and after 40 min, it was defined as the fatigue awakening phase. The combination of background music and wake-up music is shown in Table 2.

**Table 1:** Test factor names and labels.

**Name**	**Mark**	**Name**	**Mark**	**Name**	**Mark**
Blank sound	B0	Blank sound stimuli	C0	Fatigue delay phase	Fa
Positive music	B1	Positive music stimuli	C1	Fatigue awakening phase	Fb
Negative music	B2	negative music stimuli	C2	Experimental time	Ti

**Table 2:** Experiment with background music and wake-up music combinations.

**Experiment group**	**Only driving noise**	**Positive music stimulation**	**Negative music stimulation**
No background music and driving noise (control group)	Test 1		
Positive background music and driving noise (experimental group 1)		Test 2	Test 3
Negative background music and driving noise (experimental group 2)		Test 4	Test 5

In the fatigue delay stage experiment, a control group with no background music and driving noise (B0) and two test groups with background music and driving noise were set. The background music of test group 1 was positive music (B1), and the background music of test group 2 was negative music (B2). This stage of the test is mainly aimed to explore the delaying effect of different emotional background music on driving fatigue. Therefore, the data (Fa0, Fa1, Fa2) in the first 40 min of the control group, experimental group 1, and experimental group 2 were compared and analyzed.

At 40 min, a certain type of music sound stimulation was given for 1 min (60 s) to wake up from fatigue, and the test entered the stage of fatigue awakening. In this stage, five tests were set. Experiment 1 was to introduce blank sound stimuli (C0) to the control group, and experiments 2–5 were to introduce positive music (C1) and negative music stimuli (C2) to experimental groups 1 and 2, respectively. This stage of the experiment mainly explored the effect of different musical stimuli on driving fatigue, so the data (Fa01 and Fb1, Fa11 and Fb1, Fa21 and Fb2) within 20 min (T1–T2, T2–T4) before and after the introduction of the stimulus were compared and analyzed. The overall experimental design is shown in Figure 2B. After 1 min of stimulation (T3), the previous sound background was restored until the end of the test. According to the test requirements, the subjects who meet the test requirements are selected, and the subjects need to fill in the subjective evaluation questionnaire before and after each test. The pre-test questionnaire mainly investigates whether the subjects are currently sleepy and irritable, and the post-test questionnaire mainly investigates whether the subjects are tired during the test, whether the music background and stimulation can relieve their fatigue and other issues.

### 3.2 Experimental objects conditions

The test requires the subjects to be between the ages of 20 and 30, the average BMI is within the normal range, and they hold a motor vehicle driver's license C1 or above, and have certain driving experience. The visual acuity or corrected visual acuity is 1.0 and above, and there is no eye disease such as color weakness or color blindness. The subjects did not take any drugs within 1 week before the test, did not drink alcohol within 24 h, and did not drink functional beverages, coffee, etc. within 12 h, and were in good physical condition. Each subject only conducts the test once a day, and it is guaranteed that they have not listened to any music within 12 h before the test, so as to ensure that the subjects perform the test in the best state every day. The subjects were required to undergo training in the use of the simulated driver before the test to ensure that they could operate the equipment proficiently during the formal test.

### 3.3 Evaluation indicators and selection of test task materials

#### 3.3.1 Selection of evaluation indicators for fatigue detection

The eye movement behavior can effectively reflect the driver's fatigue level, and the detection method is convenient and will not interfere with the driver's behavior. At present, many scholars' studies have shown that blinking frequency and pupil diameter have high validity and application value as evaluation indicators of driving fatigue eye behavior (Wang and Li, 2015; Ma et al., 2014; Qi, 2021). Therefore, the Pupil Diameter Variation Coefficient (PDVC) and the Blink Rate Per Second (BR) in eye movement behavior were selected as the evaluation indicators of fatigue detection in this experiment.

(1) Pupil Diameter Variation Coefficient

PDVC is the ratio of the pupil diameter standard deviation to the mean of pupil diameter in the time interval, that is, the pupil fatigue rate, and its calculation formula is:


(1) $$C_{v}=\frac{\sigma }{\bar{\mu }}\times 100$$

Where is:

*C*_*v*_: Pupil Diameter Variation Coefficient, PDVC;

σ: the pupil diameter standard deviation;

$\bar{\mu }$: the mean of pupil diameter.

(2) The Blink Rate Per Second

The Blink Rate Per Second (BR) is the ratio of the total number of blinks in the time window to the duration of the time window. The BR calculation formula in the *i*-th time window is:


(2) $$\begin{array}{l} BR_{i}=\frac{BT_{ei}-BT_{si}}{T_{br}},BT_{i}\neq 0,i=1,2,...,n \end{array}$$

Where is:

BT_ei_: The total number of blinks at the end of the *i*-th time window;

BT_si_: The total number of blinks at the start of the *i*-th time window;

T_br_: The calculation time window size of BR, the optimal calculation time window of blink.

#### 3.3.2 Music clip material selection

The music materials required for the experiment are mainly selected from NetEase Cloud Music, QQ Music, and Xiami Music. First select the names of “Happy and Emotional” and “Sad and Emotionally Nervous” from the NetEase Cloud Music playlist as alternative music playlists, and then compare the songs with those in similar playlists of QQ Music and Xiami Music. Then select songs with higher repetition rate as alternative experimental task materials. Studies Yang (2016) have shown that music rhythm will affect the fatigue accumulation rate, so the songs whose music rhythm does not conform to 110 bpm (±5 bpm) are eliminated. Perform emotional semantic analysis on the lyrics of the remaining songs, so that they are divided into two categories: “positive emotions” and “negative emotions,” and the songs that do not meet the above two categories of emotions are eliminated again, and the remaining songs constitute the music material library used in the final experiment. In addition, ensure that the total duration of each music is not less than 200 min to avoid repeated playback of songs in the test. Four pieces of music of each emotional type were selected and 60 s were intercepted as the music awakening stimulation material. The blank sound stimulus was to remove the driving noise of the control group. The rest of the music and driving noise together constitute the experimental sound background, all songs are played randomly in random order, and the loudness is set at 70 dB (Hu et al., 2017).

### 3.4 Test equipment

**Figure 3:**
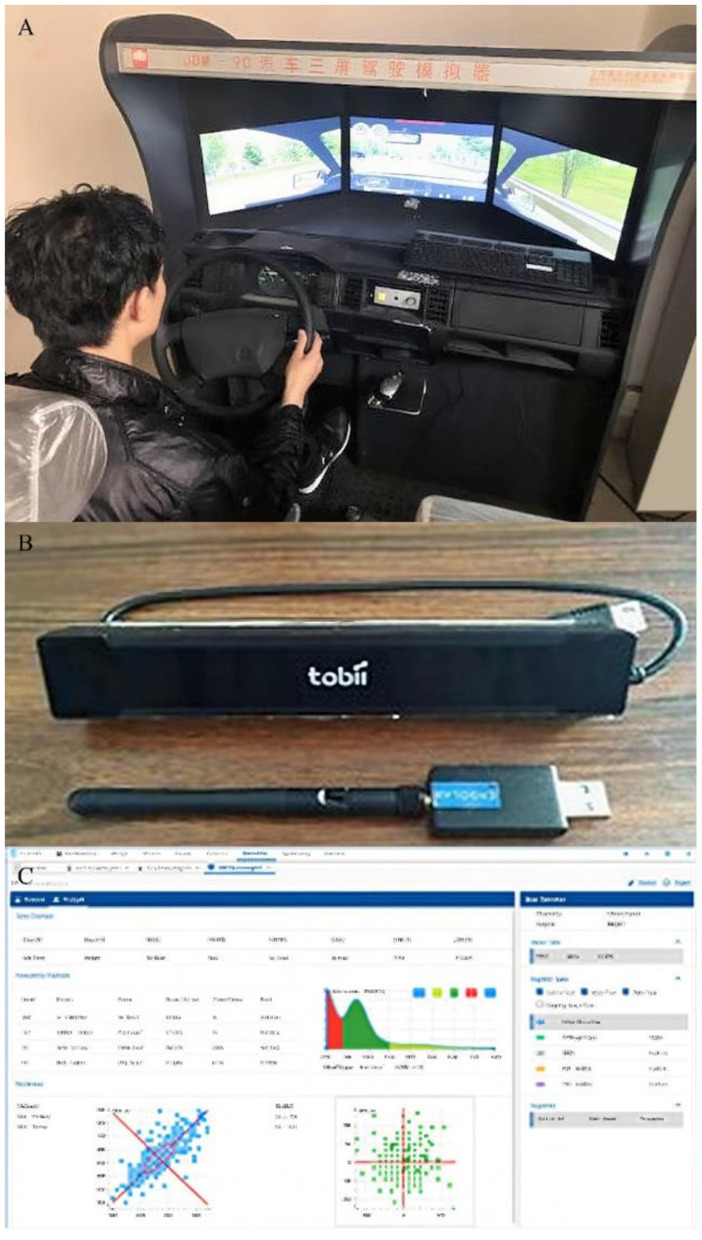
**(A)** Vehicle simulation driving platform; **(B)** eye tracker of Tobbi; **(C)** ErgoLab software.

It can be seen from the experimental design that the test equipment needs to meet the purposes of driving fatigue formation, music background and stimulation playback, and fatigue data collection. Therefore, for safety reasons, this test refers to other scholars' test methods (Zhang et al., 2010; Chen, 2017), and uses JDM-9C to simulate driving. The device is a simulated driving platform, and the equipment is shown in Figure 3A. The control layout and control method in the cab of the simulated driver are completely designed according to the real vehicle. Fatigue data was collected using the Tobii X2-30 eye tracker (as shown in Figure 3B) and the supporting ErgoLab software (as shown in Figure 3C).

The sound material playback is completed by a set of independent computer and audio equipment. In addition, in order to ensure that the test sound environment standards are consistent, the decibel meter is used to measure the volume of the test background sound according to the national standard, the cumulative percentage of the test sound is set to 75%, and the non-variable factors such as the loudness of the driving sound of the motor vehicle are controlled at within 65 dB.

## 4 Experimental procedures

**Figure 4:**
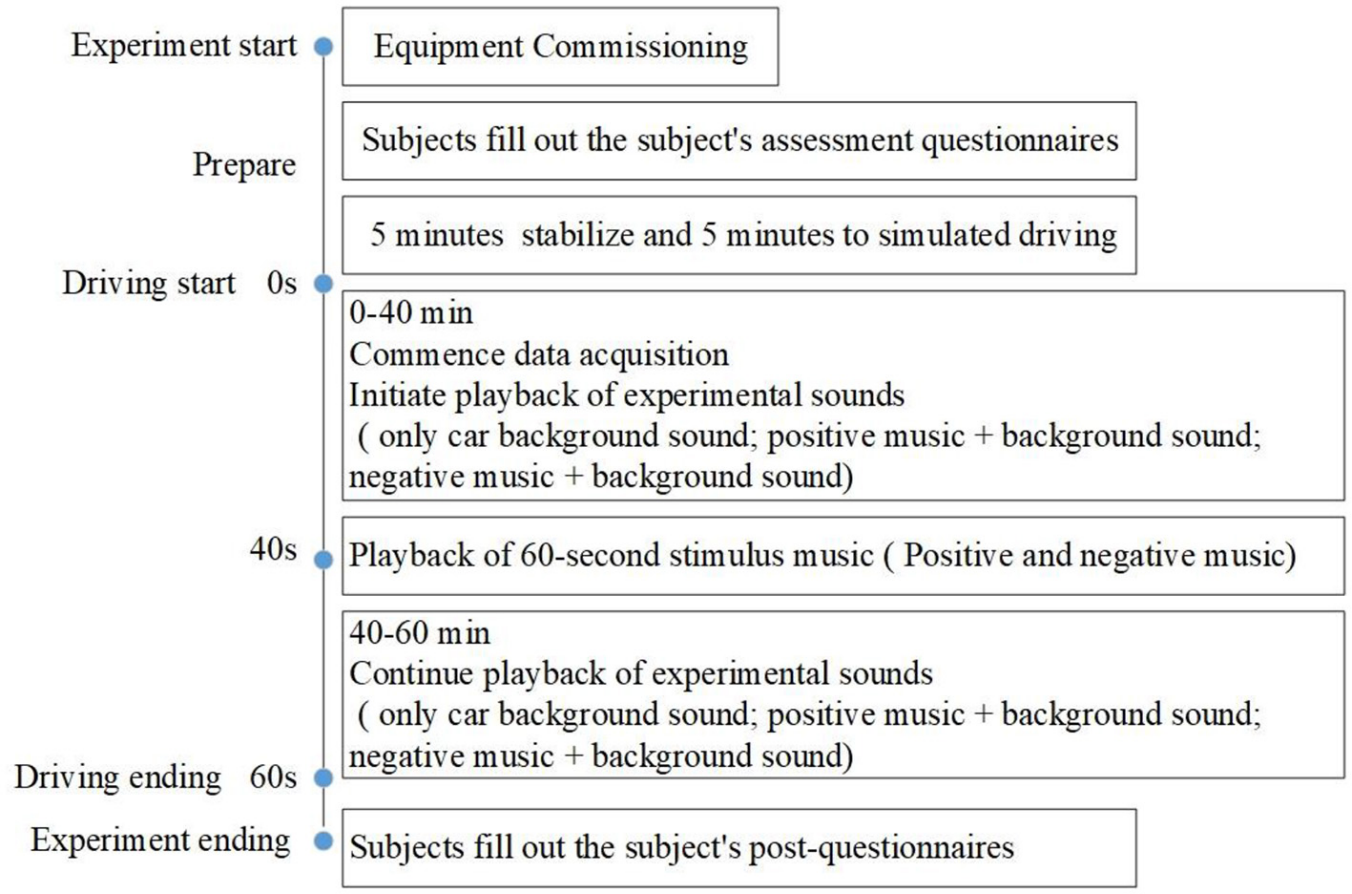
Experiment process.

**Figure 5:**
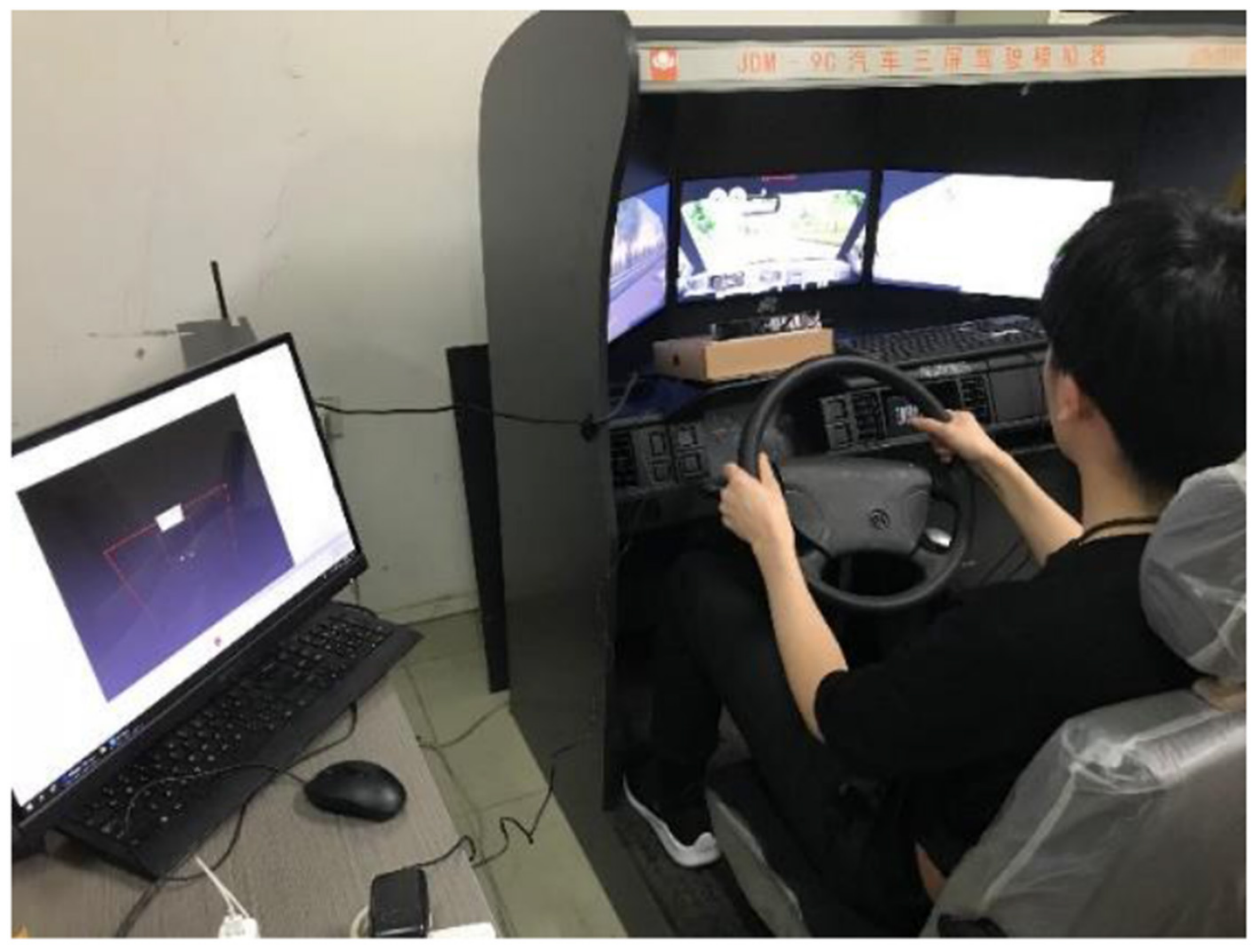
Experiment scenario.

According to the subject selection kit, 12 subjects were selected to participate in the experiment. Equipment operation training was given to each subject 1 day before the experiment. The experiment was carried out during the time when people were prone to sleepiness from 13:00 to 18:00 every day (Van Der Zwaag et al., 2012). Each subject needs to conduct five experiments (Experiment 1–Experiment 5), each time is about 60 min. Before each experiment, the subject filled out the subject's assessment questionnaire, then took 5 min even breathing to stabilize their emotions, and 5 min simulated driving to adapt to the operation. Ensuring that all the experimental equipment was working properly, the formal experiment was started. When the test reaches 40 min, a sound stimulus for 1 min is automatically given. And when the test went on for 60 min, the test was completed. After the test was completed, the subjects filled in the post-test subjective evaluation questionnaire. The specific test process is shown in Figure 4. Each subject was asked to complete four trials in the experimental group and one trial in the control group. Each subject only completed one trial per day, and the specific content is randomly selected from the four trials in the experimental group. Each subject was required to complete all five trials within 1 week. The test scene is shown in Figure 5.

## 5 Test data processing and analysis

### 5.1 Subjective questionnaire analysis

There were 60 questionnaires before the experiment and 60 questionnaires after the experiment. A total of 120 questionnaires were collected, all of which were valid questionnaires. The results of the subjective questionnaire before the test showed that all the subjects were awake before the test, without dizziness, fatigue, drowsiness and other states. Statistical analysis was carried out on the items with a higher proportion of the survey results after the test, and the results are shown in Table 3.

**Table 3:** The time period during which the subject felt fatigue.

**Questionnaires**	**Result**	**Accounting**
Whether the subject felt tired during the experiment	Yes	96.7%
Time to fatigue onset in driving	Within 30 min	81.66%
What is considered the type of music listened to	Enable judging the emotional type of music	89.59%
Whether fatigue has improved after music stimulation	Yes	77.08%

It can be seen from the above table that the subjects will feel fatigued regardless of whether there is background music in the sound background. It shows that the introduction of background music during driving cannot improve the fatigue caused by long-term driving, and people will still gradually develop fatigue. Most of the time points of fatigue appeared within 30 min. Therefore, it is considered objective and practical to judge that the subjects enter the driving fatigue state when the test is carried out to 40 min. In general, the accuracy rate of the subjects in judging the emotional type of music is 89.59%, so it is believed that the music material selected in the experiment basically meets the requirements of emotional classification. Thirty-seven people thought that driving fatigue was improved after music stimulation, accounting for 77.08%, which indicated that the introduction of music stimulation could effectively improve driving fatigue.

### 5.2 Analysis of eye movement behavior data

To avoid the influence of unstable state on the results at the beginning of the experiment, the data of the first 5 min were excluded. In order to reduce the influence of data fluctuation on the test results, 5 min was selected as the fixed analysis time interval for the test. The average value of all time intervals in a certain time period under a certain indicator is used as the score value of the indicator in this interval, so as to ensure that all indicators can be analyzed and compared in the same time dimension. The results of the overall analysis of the data show that the data are normally distributed.

#### 5.2.1 PDVC analysis under different monotonous sound backgrounds

##### 5.2.1.1 Fatigue delay phase

**Figure 6:**
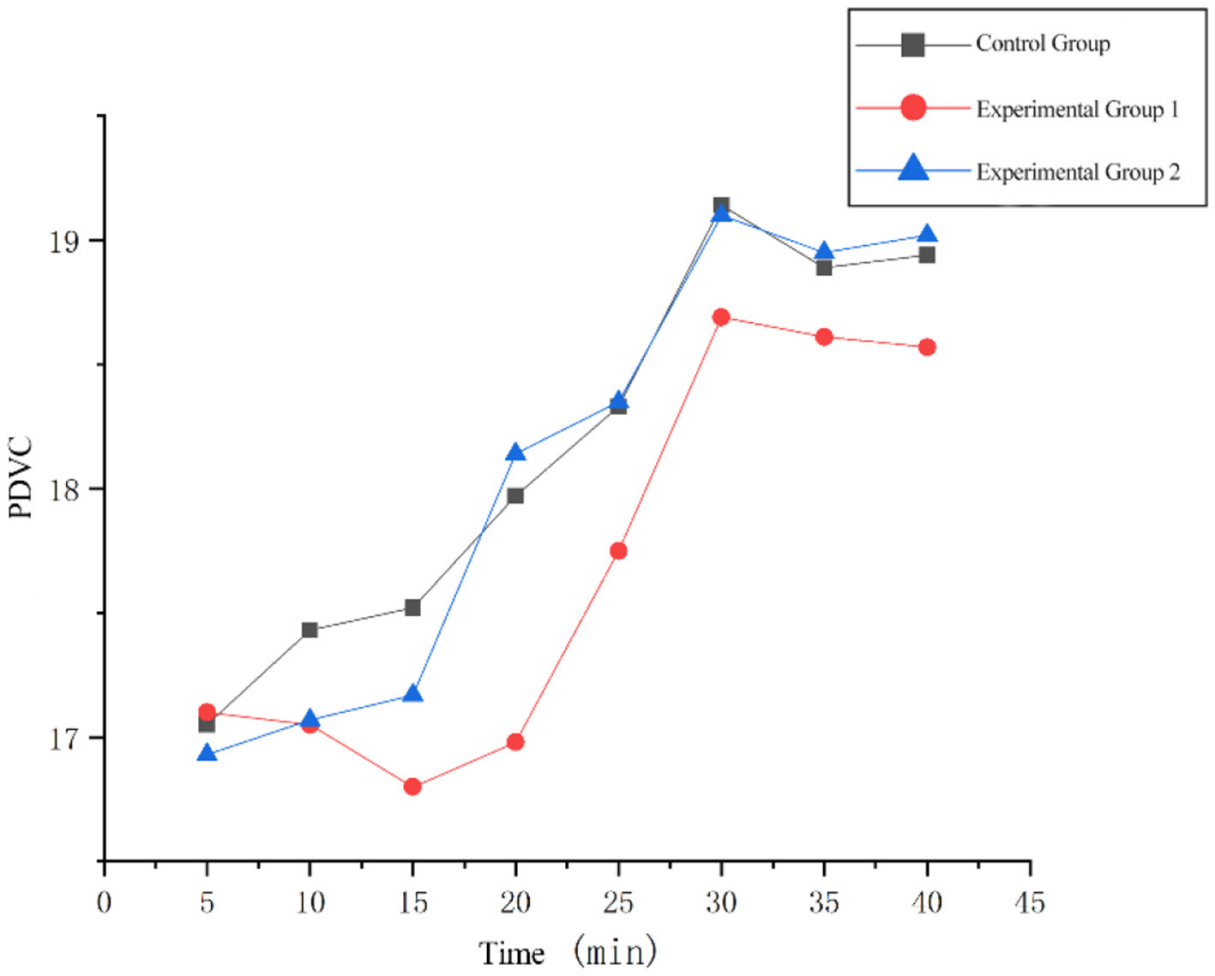
Changes of PDVC during the fatigue delay phase.

Repeated-measures ANOVA were performed on the PDVC during the fatigue delay phase. The sphericity test result *P* = 0.093, which satisfies the assumption of spherical distribution (*P* > 0.05), and the within-subject effect test result is *F* = 12.106, *p* = 0.001 < 0.05. Therefore, there was a significant statistical difference between the two experimental groups and the control group. Paired sample *t*-test was performed on PDVC. The results showed that there was a significant difference between the experimental group 1 and the control group (*t* = 4.074, *p* = 0.005 < 0.05); the difference between the experimental group 2 and the control group was not significant (*t* = 2.368, *p* = 0.055 > 0.05); the average value of PDVC in the fatigue delay stage was calculated by segment, and the 40 min PDVC change diagram was drawn, as shown in Figure 6.

a. The three groups of data overall exhibited an upward trend. This indicates a weakening of autonomic nervous system control over pupil stability due to increased cognitive load, accumulated fatigue, or declining attentional regulation. Generally, the longer the driving time, the more severe the fatigue accumulation, which aligns with objective experience.
b. The control group (no background music) showed a gradual upward trend after 15 min, reflecting the natural accumulation of fatigue as the task duration increased, leading to a progressive decline in the stability of autonomic nervous control over the pupils. A slight alleviation of fatigue appeared after 30 min, suggesting the body began adapting to the current workload.
c. Experimental Group 1 (positive music group) displayed a certain degree of decline at 10–15 min, indicating that under positive music stimulation, a brief period of focused attention or effective mobilization of cognitive resources temporarily suppressed the upward trend of the variation coefficient. It subsequently maintained a significant upward trend, showing that after a brief compensatory period, fatigue or cognitive load continued to increase, exacerbating the instability of autonomic nervous regulation. Overall, the fatigue level was lower than in the no-background-music and negative music groups, demonstrating that positive music can delay the onset of fatigue and reduce its severity.
d. Experimental Group 2 (negative music group) showed a significant rise at 15 min, suggesting that under negative music stimulation, cognitive load or fatigue significantly increased at this point, leading to a marked decline in neural regulatory stability, though the fatigue level was slightly lower than the no-background-music group. The upward trend slowed at 20 min, indicating the body entered a relative plateau phase and partially adapted to the current load. It then maintained a clear upward trend again at 25 min, showing that fatigue or load surpassed a critical threshold after the plateau phase, further intensifying regulatory instability. Fatigue reached its peak at 30 min, comparable to the level observed under no-background-music driving. This indicates that negative music can reduce fatigue in the short term but cannot delay its onset; after 25 min, it no longer has any mitigating effect.
e. All three groups reached their highest point at 30 min, signifying that overall fatigue or cognitive load peaked and neural regulatory capacity was at its most unstable state, consistent with questionnaire results. After 30 min, the values declined and stabilized, reflecting the body entering a deep fatigue stage. Here, neural resources become depleted, or the body enters a kind of “giving-up” state, where regulatory effort decreases, and variation paradoxically appears “stable” due to low-level activity.

##### 5.2.1.2 Fatigue awakening phase

Paired samples *T*-test was performed on the PDVC data of 20 min before and after fatigue awakening in Experiments 1–5. The results showed:

(1) There was a significant difference between before and after in test 1 (*t* = −0.877, *p* = 0.04 < 0.05);
(2) There was a significant difference between before and after in test 2 (*t* = 2.032, *p* = 0.049 < 0.05);
(3) There was no significant difference between before and after in test 3 (*t* = −0.427, *p* = 0.672 > 0.05);
(4) There was a significant difference between before and after in test 4 (*t* = 2.569, *p* = 0.014 < 0.05);
(5) There was no significant difference between before and after test 5 (*t* = −0.399, *p* = 0.692 > 0.05).

**Figure 7:**
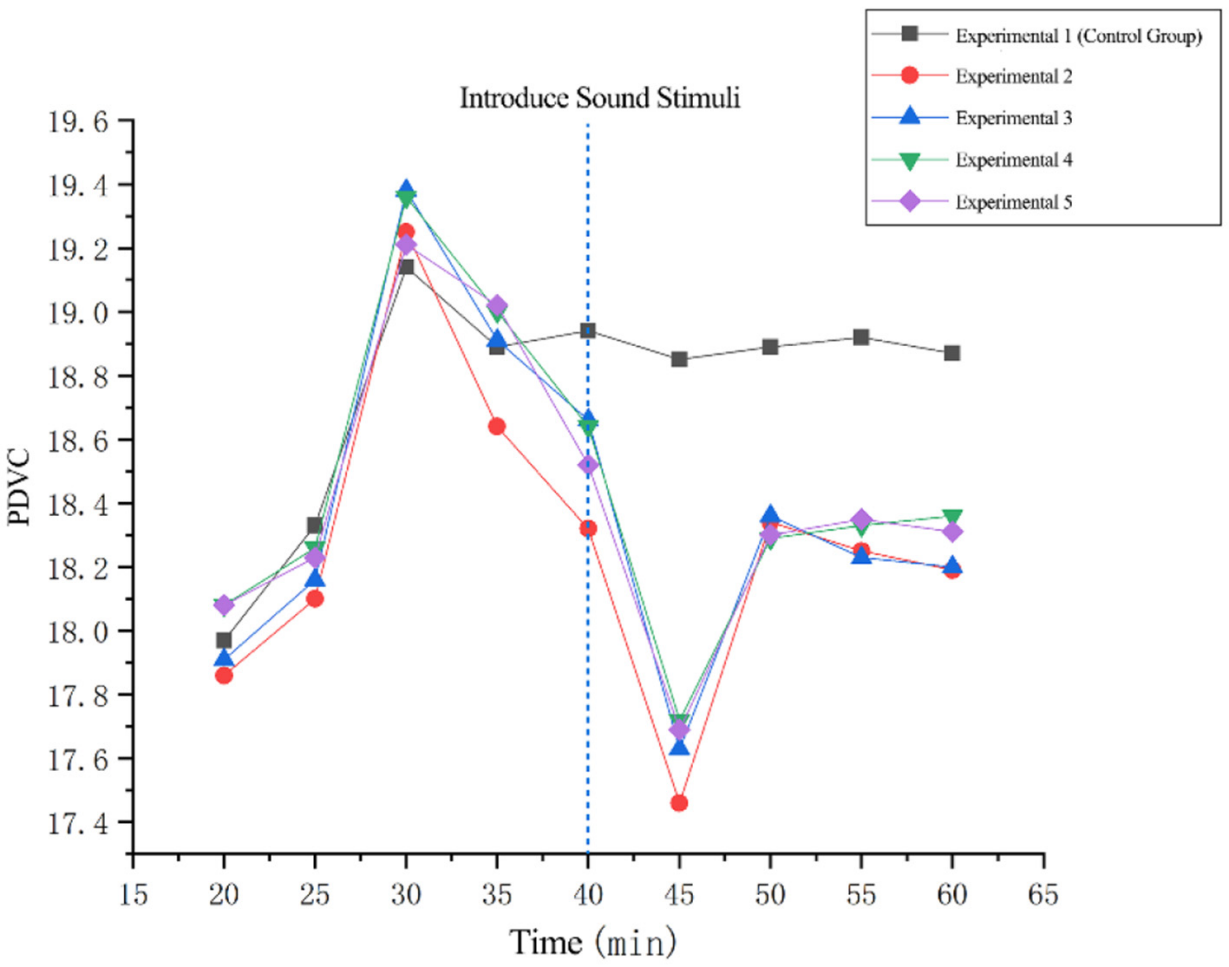
PDVC before and after arousal stimulation.

At the same time, when comparing Experiment 2 with Experiment 4, Experiment 4 (*p* = 0.014 < 0.05) had a more significant difference than Experiment 2 (*p* = 0.049 < 0.05). Figure 7 shows the change in the PDVC before and after the awakening stimulus is drawn.

a. All five groups of data showed a declining trend at 40 min. For the no-music background group (control group), this decline occurred because after sustained fatigue, the body entered a brief compensatory period or resource reorganization phase. The autonomic nervous system attempted to maintain basic functions by reducing regulatory volatility (manifested as a decrease in PDVC). The declining trend was particularly pronounced in the other four experimental groups compared to the control group. This suggests that the introduced arousal stimuli likely accelerated or amplified this compensatory/adjustment process, making the pupillary regulation in the experimental groups appear more “focused” or stable at this point. It indicates that all music stimuli produced an arousal effect, with the switch from negative to positive music background being the most effective. Under fatigue, prolonged negative music-induced neural inhibition (decreased dopamine/norepinephrine levels) exacerbates cognitive inertia. At this point, switching to positive music induces a strong emotional polarity reversal (negative to positive), activating the limbic system (amygdala, nucleus accumbens) and the locus coeruleus (LC). This triggers a burst release of dopamine and norepinephrine, significantly enhances sympathetic tone, and reactivates prefrontal cognitive functions. Consequently, pupillary regulation volatility (pupil variation coefficient) increases sharply, efficiently disrupting the fatigue steady state. Conversely, switching from positive to negative music results in a milder effect. Due to partial desensitization of neurotransmitter receptors, negative music primarily induces unidirectional inhibition (increased parasympathetic dominance and elevated GABA, the core inhibitory neurotransmitter in the brain). This lacks sufficient neural conflict signals to break through the existing arousal plateau, leading to gentler changes in pupillary regulation (smaller PDVC increase) and a weaker fatigue-arousing effect. The core of this asymmetric mechanism lies in the intensity of emotional contrast: the “providing timely help” switch (negative to positive) generates a super-compensatory effect through intense neurochemical and emotional reversal, while the “diminishing returns” reverse switch (positive to negative) fails to fully trigger the compensatory rebound of the nervous system, limiting its effectiveness.
b. All data showed an upward trend at 45 min. This marks the end of the brief compensatory period, where accumulated fatigue or cognitive load regains dominance, and the stability of autonomic nervous system control over the pupils decreases again (PDVC rises). This likely corresponds to the waning effect of the arousal stimulus or a new wave of fatigue impact. The upward trend was extremely pronounced in all four experimental groups compared to the no-music background group (control group), signaling the rapid decline of the music stimulus effect. This indicates that introducing brief music stimuli during fatigue can induce arousal, but the duration of its effect is limited. All data tended toward stable changes after 50 min, entering a fatigue plateau phase. Compared to the no-music stimulus, the level of fatigue following music stimulation was significantly lower. This suggests that after the intense initial arousal phase, the music stimuli continued to provide a fatigue-alleviating effect even during the subsequent plateau phase.

#### 5.2.2 Analysis of BR under different monotonous sound backgrounds

##### 5.2.2.1 Fatigue delay phase

**Figure 8:**
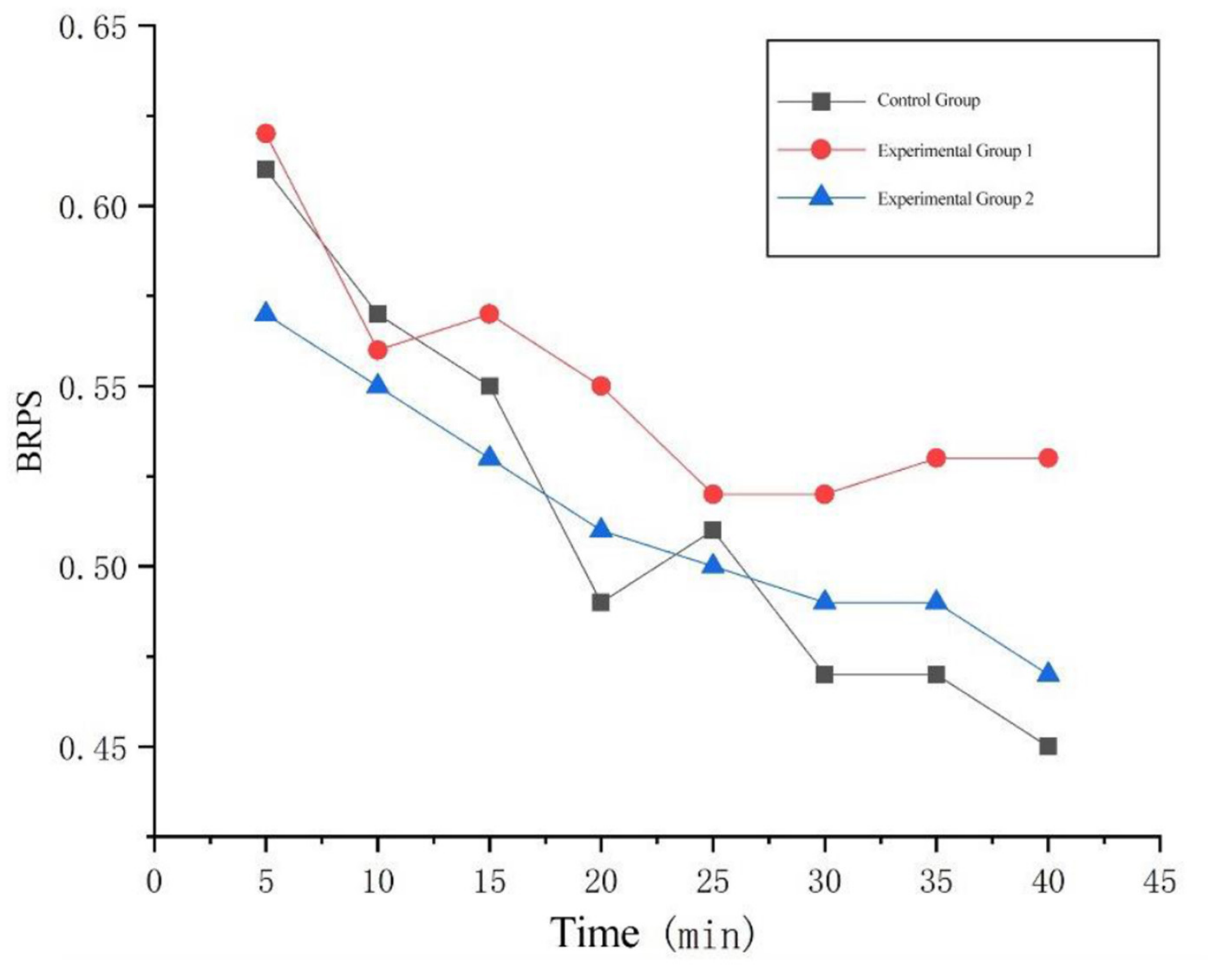
Changes in mean blink rate during fatigue delay phase.

Repeated measures ANOVA were performed on BR, and the results of spherical test showed *p* = 0.130 < 0.05, which satisfied the assumption of spherical distribution (*P* > 0.05). The results of within-subject effect test were *F*_(2, 14)_ = 11.050 (*p* = 0.001 < 0.05). Therefore, there is a significant difference between the two experimental groups and the control group. Then the paired sample *T*-test was performed on the BR data of the experimental group and the control group. The results showed that there was a significant difference between the experimental group 1 and the control group (*t* = 3.130, *p* = 0.017 < 0.05). There was no significant difference between the experimental group 2 and the control group (*t* = −0.146, *p* = 0.888 > 0.05); there was a significant difference between the experimental group 1 and the experimental group 2 (*t* = 6.416, *p* = 0.000 < 0.05). The BR in the fatigue delay stage was averaged, and the BR change diagram in the early 40 min of the test was drawn, as shown in Figure 8.

a. The data from the three groups overall exhibited a downward trend during the first 40 min. The decline in average blink rate typically reflects two physiological states: (1) Blink inhibition due to sustained allocation of attentional resources (common in cognitive tasks); (2) Weakening of neural drive to the orbicularis oculi muscle caused by accumulated fatigue. The overall downward trend indicates that driving fatigue intensified with prolonged time. Among them, the no-background-music group (control group) showed the greatest degree of decline, suggesting that this unintervened group experienced the fastest depletion of cognitive resources and the most significant mental fatigue.
b. Compared to the no-background-music group, the data from the two groups with music stimulation (Experimental Group 1) were generally at a higher level. This indicates that music may partially counteract the blink inhibition induced by fatigue by sustaining the activity of the locus coeruleus-norepinephrine (LC-NE) system, maintaining the blink rate at a relatively higher level, reflecting better sustained alertness. The no-background-music group (control group) and the positive-background-music group (Experimental Group 1) exhibited significant fluctuations during their decline. These fluctuations may stem from: (a) A tug-of-war between fatigue and cognitive compensation (e.g., brief recovery of attention causing a temporary rebound in blink rate); (b) Instability in neural regulation leading to fluctuations in motor control, synchronizing with the mechanism of autonomic nervous fluctuation during the PDVC increase phase. The negative-background-music group (Experimental Group 2) showed a steady downward trend, indicating that the intervention in this group failed to effectively activate compensatory mechanisms. Neural resources entered a state of linear exhaustion, and while the stability of the oculomotor control system remained high, it functioned at a low level (similar to the plateauing mechanism observed in the later stages of PDVC).

##### 5.2.2.2 Fatigue awakening phase

**Figure 9:**
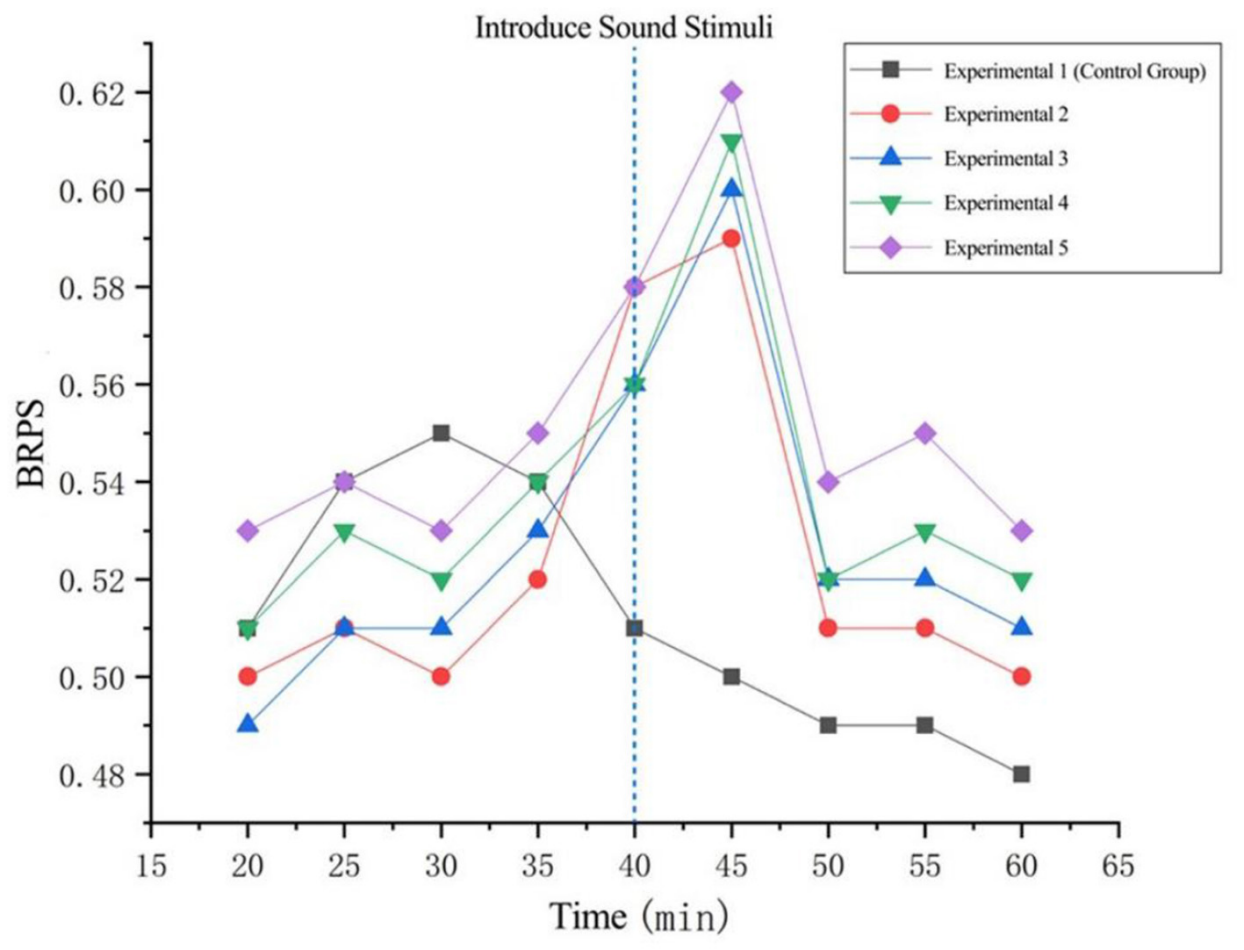
Changes of BRPS before and after arousal stimulation.

A paired sample *T*-test was conducted on the average blink rate data of 20 min before and after fatigue awakening in Experiments 2–5. The results showed that there was a significant difference in the data before and after Experiment 1 (*t* = 1.532, *p* = 0.041 < 0.05); there was a significant difference in the data before and after Experiment 2 (*t* = −0.632, *p* = 0.035 < 0.05); there was no significant difference in the data before and after Experiment 3 (*t* = 0.388, *p* = 0.700 > 0.05); there was a significant difference in the data before and after Experiment 4 (*t* = −1.725, *p* = 0.009 < 0.05); and there was no significant difference in the data before and after Experiment 5 (*t* = 0.242, *p* = 0.810 > 0.05). Meanwhile, compared with Experiment 2, the difference in the average blink rate before and after Experiment 4 (*p* = 0.035 < 0.05) was more significant than that of Experiment 2 (*p* = 0.009 < 0.05). The changes in the average blink rate before and after the awakening stimulus are shown in Figure 9.

The average blink rate across all four experimental groups began to rise from the 30-min mark, indicating progressively worsening fatigue accumulation. The upward trend became more pronounced during the 40–45 min period. This is because the music stimulus introduced at the 40-min mark brought about a significant impact. It suggests that the effectiveness of the arousal intervention reached its peak at this time: (1) Burst release of neurotransmitters from the locus coeruleus directly excited motor neurons of the levator palpebrae superioris muscle; (2) The dopaminergic pathway transiently enhanced the disinhibition of the orbicularis oculi muscle by the basal ganglia, promoting a rebound in blink frequency. As can be seen from the graph, whether with negative or positive background music, changing the music tempo or tracks achieved a certain level of arousal effect. After 45 min, all experimental groups exhibited a distinct downward trend, indicating that after a period of stimulation, the neurochemical effects of the arousal stimuli began to attenuate, while cumulative fatigue regained dominance, and inhibitory control over blink movements by the prefrontal-thalamic pathway was reinforced.

## 6 Result and discussion

The paired *T*-test results of the fatigue delay stage data of the three experimental groups showed that there were significant differences between Experimental Group 1 and the control group, as well as between Experimental Group 2 and the control group. That is, compared with no music background or negative music background, positive music background had a certain alleviating effect on driving fatigue.

From the data graph, it can be found that the fatigue depth of the subjects in the positive music background was shallower than that of the subjects in the negative music background and no background music. This is because music can reduce the frequency in the low-frequency band of heart rate variability and increase the frequency in the high-frequency band, thereby enhancing the excitability of the parasympathetic nerve and alleviating job fatigue (Zhang et al., 2017). However, the subjective questionnaire results showed that the subjects were still in a fatigued state after the experiment. A comprehensive analysis indicated that when different emotional background music was introduced at the beginning of driving, fatigue accumulation would still occur after long-term driving, but the fatigue depth of the subjects in the positive music background was shallower, that is, the time for deep fatigue to occur would be delayed. Some studies have shown (Zhao et al., 2022) that after long-term driving, if drivers stop driving and listen to soothing music during the fatigue recovery stage, the fatigue relief effect is obvious. This is inconsistent with the results of this experiment. The reason for this phenomenon may be related to whether the subjects are in a monotonous working state. When drivers are in a monotonous working state, the degree of fatigue accumulates over time. Although music can relieve fatigue, the degree of relief during the 40-min continuous driving process may be less than the degree of fatigue accumulation, thus making the fatigue relief effect of driving not obvious. It can be seen that positive music can reduce the depth of fatigue to a certain extent, but deep fatigue will still occur after long-term driving.

The paired *T*-test results of the coefficient of variation of pupil diameter and average blink rate 20 min before and after the introduction of fatigue arousal stimuli in Experiments 2–5 all showed that there were significant statistical differences before and after Experiments 2 and 4, and the difference before and after Experiment 4 was more significant. There were no significant differences before and after Experiments 3 and 5.

The graphs of pupil diameter variation and average blink rate also showed that the data changed significantly at 40 min. The comprehensive analysis indicated that introducing positive music in both negative and positive music backgrounds could effectively alleviate driving fatigue, and the fatigue arousal effect was better when positive music was introduced in a negative music background, which was consistent with the results of the subjective evaluation questionnaire. This situation might be due to the strong rhythm of positive music, which could make drivers more alert (Sahayadhas et al., 2012), thereby increasing the alertness and attention of the subjects, activating the locus coeruleus-norepinephrine system, and causing it to release neurotransmitters that act on the dilator muscle, resulting in pupil dilation (Yang et al., 2020), and a significant change in the coefficient of variation of pupil diameter. In addition, positive music might be more likely to cause the subjects' startle reflex, causing rapid contraction of the orbicularis oculi muscle and increasing the blink rate. Introducing positive music in a negative music background might have a stronger stimulating effect due to the large difference in music types before and after, making the subjects' eye movement physiological responses more obvious and thus achieving a better arousal effect. Through the data change graph, it can be found that the significant change in the data after 40 min did not last long and returned to the state before the stimulation within a few minutes, which was consistent with the findings of Mao et al. (2010). Therefore, this kind of music stimulation is only suitable for rest prompts or alerts to improve driving alertness and cannot be used as a solution to alleviate long-term driving fatigue.

## 7 Conclusion

The study investigates the impact of different musical backgrounds on driving fatigue and the effectiveness of corresponding arousal countermeasures. Through conducting a simulated driving control experiment, the changes in fatigue levels under different sound background driving conditions and the fatigue arousal effects of different types of music stimulation were analyzed. The research conclusions of this paper are as follows.

### 7.1 Positive music delays driving fatigue

When music stimulation is introduced as the driving sound background at the beginning of driving, the degree of fatigue under the background of positive music is relatively shallow, that is, music has a delaying effect on the generation of fatigue. The design of fatigue awakening products can use positive music as the background music for vehicle driving to delay the fatigue caused by long-term driving of users.

### 7.2 Positive music relieves driving fatigue effectively

Introducing positive music stimulation as a countermeasure for driving fatigue has certain effects, especially when it is introduced against a background of negative music, the effect is more significant. Positive music can be used as a fatigue awakening stimulus to relieve driver fatigue in a short time and serve as an audible prompt for drivers to stop and rest when fatigue is detected.

This paper only conducted a 60-min simulated driving test and a 1-min sound stimulation impact study. In the future, the driving time can be further extended or the duration of sound stimulation can be changed to explore whether the duration will affect the degree to which music background and stimulation alleviate driving fatigue. In addition, the above experiments did not consider the influence of personal factors such as the subjects' preferences for music types. Future research can further improve the driving study by controlling multiple factors to make it more scientific.

## Data Availability

The raw data supporting the conclusions of this article will be made available by the authors, without undue reservation.
